# Harnessing the Plasma Proteome to Mirror Current and Predict Future Cardiac Remodeling After Myocardial Infarction

**DOI:** 10.1007/s12265-022-10326-w

**Published:** 2022-10-05

**Authors:** Upendra Chalise, Mediha Becirovic-Agic, Jocelyn R. Rodriguez-Paar, Shelby R. Konfrst, Sharon D. B. de Morais, Catherine S. Johnson, Elizabeth R. Flynn, Michael E. Hall, Daniel R. Anderson, Leah M. Cook, Kristine Y. DeLeon-Pennell, Merry L. Lindsey

**Affiliations:** 1 Department of Cellular and Integrative Physiology, Center for Heart and Vascular Research, University of Nebraska Medical Center, Omaha, NE 68198 USA; 2 Research Service, Nebraska-Western Iowa Health Care System, Omaha, NE 68198 USA; 3 Eppley Institute for Research in Cancer and Allied Diseases, University of Nebraska Medical Center, Omaha, Nebraska 68198 USA; 4 Department of Physiology and Biophysics, University of Mississippi Medical Center, Jackson, MS 39216 USA; 5 Division of Cardiovascular Medicine, Department of Internal Medicine, University of Nebraska Medical Center, Omaha, NE 68198 USA; 6 Department of Pathology and Microbiology, University of Nebraska Medical Center, Omaha, NE 68198 USA; 7 Department of Medicine, Division of Cardiology, Medical University of South Carolina, Charleston, SC 29425 USA; 8 Research Service, Ralph H. Johnson Veterans Affairs Medical Center, Charleston, SC 29401 USA; 9 School of Graduate Studies and Research, Meharry Medical College, 1005 Dr DB Todd Jr Blvd, Nashville, TN 37208 USA; 10 Nashville VA Medical Center, Nashville, TN 37212 USA

**Keywords:** Wound healing, Infarct wall thinning, Left ventricular dilation, Proteomics, Echocardiography, Outcomes, ApoA1, TIMP-1

## Abstract

To identify plasma proteins that mirror current and predict future remodeling after myocardial infarction (MI), we retrospectively interrogated plasma proteomes of day (D)0 control (*n* = 16) and D3 MI (*n* = 15) from C57BL/6 J mice (20 ± 1 months). A total of 165 unique proteins were correlated with cardiac physiology variables. We prospectively tested the hypothesis that candidates identified retrospectively would predict cardiac physiology at an extended timepoint (D7 MI) in a second cohort of mice (*n* = 4 ± 1 months). We also examined human plasma from healthy controls (*n* = 18) and patients 48 h after presentation for MI (*n* = 41). Retrospectively, we identified 5 strong reflectors of remodeling (all *r* ≥ 0.60 and *p* < 0.05). Prospectively, ApoA1, IgA, IL-17E, and TIMP-1 mirrored current and predicted future remodeling. In humans, cytokine-cytokine receptor signaling was the top enriched KEGG pathway for all candidates. In summary, we identified plasma proteins that serve as useful prognostic indicators of adverse remodeling and progression to heart failure.

**Graphical Abstract:**

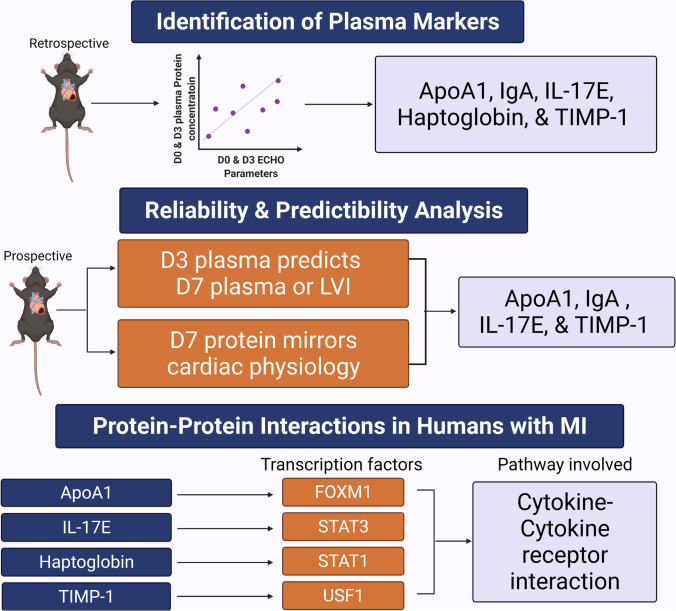

**Supplementary Information:**

The online version contains supplementary material available at 10.1007/s12265-022-10326-w.

## Introduction

Heart failure with reduced ejection fraction has a high 5-year mortality rate of 50% and is irreversible in progression [1–4]. In about 80% of heart failure cases, myocardial infarction (MI) is the underlying etiology. MI induces robust infarct wall thinning and dilation of the left ventricle (LV) over the first week in mice [5–8]. As a response to MI, cardiac wound healing initiates early inflammation to clear necrotic debris followed by fibroblast activation to secrete extracellular matrix needed to form the infarct scar, a process similar in both mice and humans [9–11]. Cardiac remodeling begins with infarct wall thinning due to cardiomyocyte necrosis, with the greatest extent of wall thinning occurring within the first 24 h of MI in the mouse model of permanent occlusion [7, 12, 13]. Wall thinning is followed by LV dilation as a feedback response to reduced ejection fraction and volume overload [14, 15]. Understanding how early events of cardiac wound healing can predict later progression to heart failure will provide prognostic indicators for at-risk patients at a time when intervention to prevent the progression to heart failure is still possible.

Plasma markers that reflect the inflammatory component of MI have been identified as predictors of future outcomes [2, 4]. These include c-reactive protein, matrix metalloproteinase (MMP)-9, myeloperoxidase, galectin-3, and neutrophil blood count, all of which have been shown in mice and in humans to predict progression to heart failure and death [16–29]. The early phase of pro-inflammation is followed by consecutive phases of anti-inflammation and repair [8, 30]. As such, inflammatory markers that are transient in expression in plasma may not hold predictive reliability at every time point of MI. Furthermore, transient expression dictates the need to measure at the right time, and times of peak expression may not be uniform across individual patients. As the response to MI is dynamic across the wound healing continuum, markers that continuously indicate and predict cardiac dysfunction over a range of times after MI would be of high clinical relevance [9, 12].

Reliable biomarkers with uniform patterns will aid in better treatment for patients with MI by providing an easier way to develop a personalized medicine strategy and assess response to therapy [31, 32]. Identification of functionally relevant biomarkers also reveals signaling mechanisms, which potentially will uncover new therapeutic targets. Here, we used data from three different proteomics platforms in mice to identify plasma markers of adverse cardiac remodeling after MI. We hypothesized that the identified plasma markers from a first cohort would reflect cardiac physiology across an extended timepoint of MI in a second cohort of mice monitored at two times. We also examined the glycoproteome of human plasma from patients with MI to identify signaling pathways associated with the identified biomarkers to provide a translational context.

## Methods

### Experimental Design

**Fig. 1:**
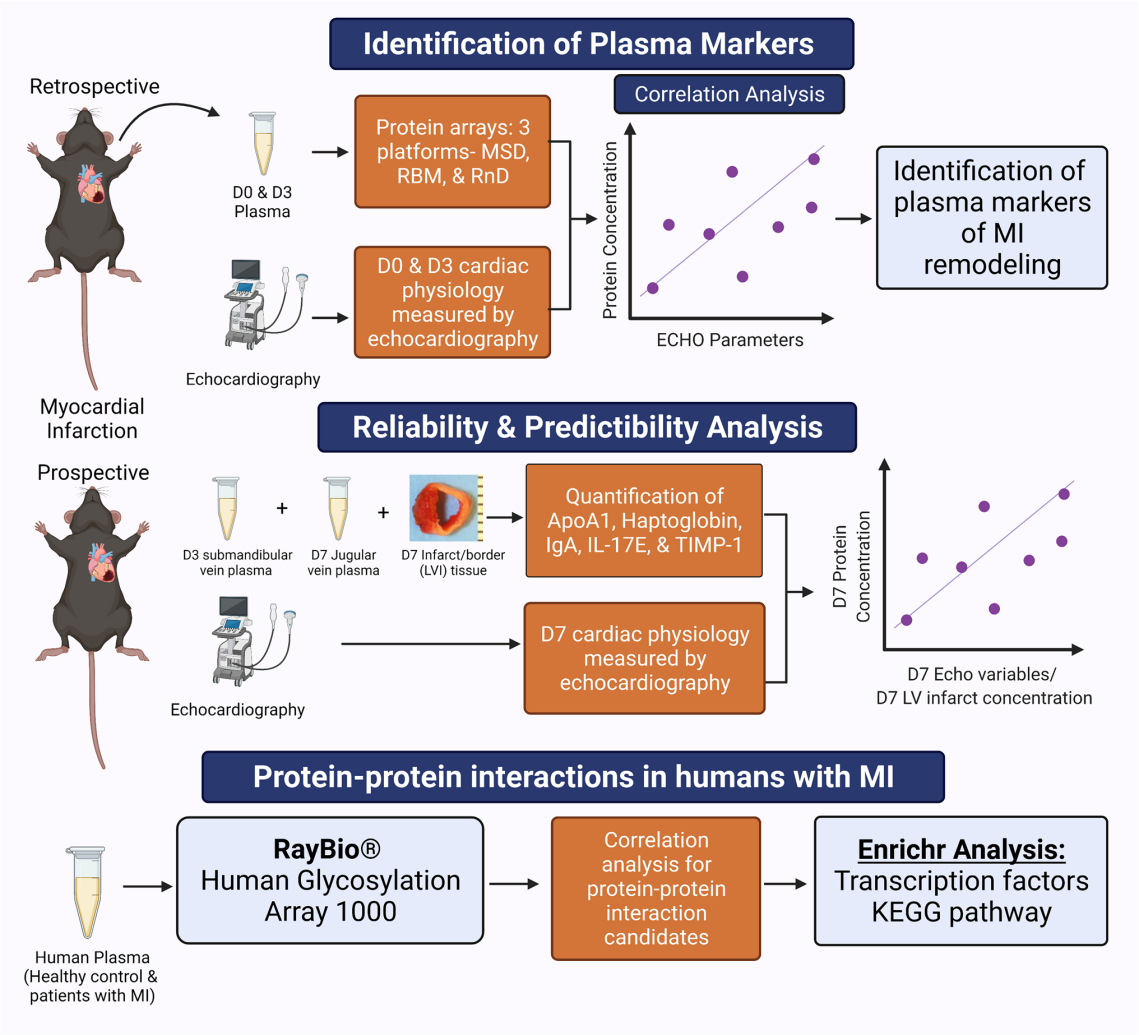
Experimental design. Created with BioRender.com

The experimental design included a retrospective study using a previously collected database and tissue bank, a prospective study using a new cohort of mice, and a human cohort (Fig. 1). All animal procedures were approved by the Institutional Animal Care and Use Committee and were conducted in accordance with the Guide for the Care and Use of Laboratory Animals published by the National Institutes of Health [33]. The human subject protocol was approved by the Institutional Review Board at the University of Mississippi Medical Center (IRB# 2013–0164). C57BL/6 J wild-type mice were selected for the study because they are the most frequently used genetic strain and recommended for studies evaluating inflammatory responses. MI day (D)3 and D7 were selected as time points of evaluation because they reflect different cardiac wound healing phases. D3 reflects the inflammatory and early anti-inflammatory phase while D7 reflects the inflammation resolution, tissue repair, and scar formation phase. We used targeted proteomics platforms that combined measured 165 targeted proteins.

### Retrospective Study

The mouse Heart Attack Research Tool (mHART) database and tissue bank consists of data combined from across MI projects published since 2007 under standard operating procedures from a single site lab [34]. For the retrospective analysis, we included data for male and female C57BL/6 J mice ranging from 14 to 25 months (average age 20 ± 1 months) were evaluated at no MI D0 (*n* = 16, 8 M/8F) or MI D3 (*n* = 15, 11F/4 M). Plasma sampling in the retrospective cohort was cross-sectional rather than longitudinal in design. The database was accessed on May 2020 by UC. The plasma analysis was originally evaluated by multi-analyte profiling using a 60-protein array (Rules-Based Medicine/Myriad). Echocardiography included examination of dimensions: end systolic dimension (ESD) and end diastolic dimension (EDD); volumes: end systolic volume (ESV) and end diastolic volume (EDV); infarct wall thickness (IWT) measured as anterior wall thickness at systole; and ejection fraction (EF). Infarct size was assessed by 1% 2,3,5-triphenyl tetrazolium chloride (TTC) staining and calculated as a percentage of the LV area that was infarcted [7, 12]. We additionally screened the banked plasma using two additional protein arrays- V-PLEX Plus Mouse Cytokine 29-plex Kit (Meso Scale Discovery (MSD), Cat. No. K15267G) and Proteome Profiler Mouse XL Cytokine Array (R&D Systems (R&D), Cat. No. ARY028) containing 111 proteins, both of which were performed according to manufacturer instructions. Combined, this yielded 165 unique plasma analytes, 6 variables of cardiac physiology, and infarct area assessed at necropsy.

### Prospective Study

A new cohort of mice (*n* = 20, 9 M/11F) ranging from 3 to 6 months of age (average age 4 ± 1 months) underwent surgery, and cardiac physiology was assessed by echocardiography as above and per established guidelines and standard operating procedures [3, 35–39]. At D3 of MI, the mice underwent serial echocardiography assessment using the VEVO 3100 (Fujifilm), and blood (100 µL) was collected from the submandibular vein and placed in a tube with 20 µL heparin (1000 USP units/ml, Fresenius Kabi, Cat No. NDC 63,323–540-15) to obtain plasma. At MI D7, the mice underwent echocardiography and were euthanized. Heparin (4 µl/g body weight) was injected intraperitoneally 5 min before euthanasia, and blood was collected from the jugular vein to obtain D7 plasma, and the LV infarct region (infarct and border, LVI) was separated from the remote region and snap frozen.

### Immunoblotting

Immunoblotting was performed according to the published guidelines [40]. Immunoblotting was performed on MI D3 and D7 plasma samples from the second cohort to validate and extend the targeted proteomic array results, and immunoblotting was performed on D7 LVI samples to examine if LVI was a source of the protein in the plasma. LV infarct protein was homogenized in TPER™ tissue protein extraction buffer (16 µl/mg LVI, Thermo Fisher, Cat. No. 78510). Protein concentration was quantified using the Nanodrop (Thermo Fisher, Cat. No. ND2000).

A total of 0.1 µL volume for plasma or 10 ug total protein for LVI was loaded onto 4–12% Criterion XT Bis–Tris precast gels (Bio-Rad, Cat. No. 345–0125) and transferred onto Trans-Blot Turbo Transfer Pack Nitrocellulose Membranes (Bio-Rad, Cat. No. 170–4159). The membranes were stained with Pierce Reversible Protein Stain Kit for nitrocellulose membranes (Thermo Scientific, Rockford, IL), and densitometry was analyzed for normalization of the LVI samples [7, 37, 41, 42]. Membranes were blocked with Blotting Grade Blocker (Bio-Rad) in 5% triphosphate buffer solution and incubated overnight with primary antibody at 4 °C followed by incubation at room temperature for 1 h with secondary antibody. Antibodies and dilutions used included ApoA1 (Abcam, Cat. No. ab227455, 1:1000), haptoglobin (Thermo Fisher Scientific, Cat. No. MA5-32,584,1:1000), IL-17E/IL-25 (R&D systems, Cat. No. MAB1399, 1:1000), and TIMP-1 (Thermo Fisher Scientific, Cat. No. MA5-13,688, 1:1000). Of note, we tried the following 4 antibodies for TIMP-1 before finding one that showed suitable specificity at the right expected molecular weight: Abcam ab38978, ab216432, and ab179580, and Epitomics 3346–1 (all at 1:1000) did not show specific binding.

For ApoA1 and haptoglobin primary antibodies, the blots were incubated with goat anti-rabbit IgG secondary antibody (Vector Laboratories, Cat. No. PI-1000, 1:5000). For IL-17E/IL-25 primary antibody, the blot was incubated with goat anti-rat IgG secondary antibody (Vector Laboratories, Cat. No. PI-9400, 1:5000). For the TIMP-1 primary antibody, the blot was incubated with horse anti-mouse IgG secondary antibody (Vector laboratories, Cat. No. PI-2000, 1:5000). Chemiluminescent images were captured using the iBright FL1000 imaging system (Thermo Fisher) and quantified using iBright analysis software 4.0.0. For the MI D3 and D7 plasma analysis, samples were volume loaded and data presented as arbitrary units. For the D7 LVI tissue analysis, the blots were normalized to total protein and data presented as normalized arbitrary units.

### IgA Isotyping Panel

IgA expression was quantified in plasma and LVI homogenates using a mouse isotyping panel (MSD, Cat. No. K15183B) per manufacturer recommendations. The plate contained 7 antibody fixed spots in each well of the isotyping panel, and each spot was linked to electrodes for signal quantification. A sulfo-tagged antibody was used with appropriate read buffer after sample incubation, and electrical signals were quantified. Plasma samples were volume loaded and reported as µg/ml, whereas LVI samples were normalized to total protein and reported as nanograms per microgram of total protein, with appropriate dilution correction for each. Plasma samples were run at 1:10,000 dilution, and LV infarct samples were run at 1:100 dilution.

### Protein–Protein Expression in Human Glycoprotein Array

A previously published human plasma glycoproteomic dataset was used for analysis of protein–protein interactions for 4 of the plasma protein candidates: ApoA1, haptoglobin, IL-17E, and TIMP-1[11]. IgA was not measured in the glycoarray. The dataset contained plasma expression of 1000 glycoproteins from healthy controls (*n* = 18) or MI patients 48 h after presentation (*n* = 41). Table 1 contains the patient characteristics. Protein–protein interactions were assessed by correlation analysis of plasma candidates in the glycoarray with the 999 other glycoproteins measured. Significant correlation (*p* < 0.05) was used for enrichment analysis [43, 44].

**Table 1:** Patient characteristics [11]

	Healthy controls (*n* = 18)	MI (48 h; *n* = 41)
Age (years)	48 (range 25–76)	56 (range 33–72)
Sex	14 women, 4 men	18 women, 23 men
Race	7 Black, 11 White	18 Black, 23 White

### Statistical Analysis and Bioinformatics

Statistical analyses were performed with GraphPad Prism 9 according to the guidelines outlined in Statistical Considerations in Reporting Cardiovascular Research [45]. For all analyses, *p* < 0.05 was considered significant. Pearson correlations were conducted for the 6 cardiac physiology variables and all 165 protein candidates. Protein candidates with strongest correlations (*r* > 0.60) in the retrospective cohort were identified as plasma reflectors and accepted for further evaluation in the prospective cohort. Unpaired *t*-test was used to analyze retrospective data in D0 and MI D3 plasma derived from different mice, and a paired t-test was used for the prospective data with MI D3 and D7 plasma derived from the same mouse. The bioinformatics tool Enrichr (https://maayanlab.cloud/Enrichr/) was used for the enrichment analysis to identify transcription factors and pathways enriched in the list of protein interactors for each of the plasma protein candidates [43, 44]. Bubble charts were created by summation of combined z-scores for each of the pathways in all 4 proteins for data visualization.

## Results

### Five Candidates in the MI D3 Plasma Proteome Mirrored Cardiac Physiology

**Fig. 2:**
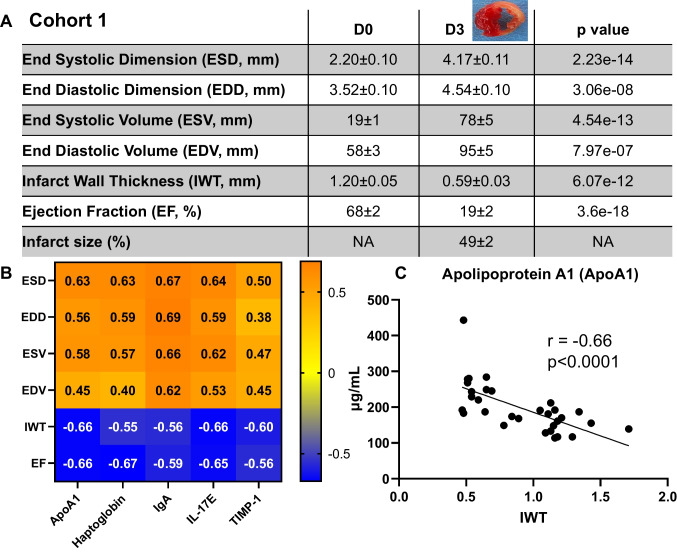
Identification of 5 proteins as reflective indicators of cardiac physiology responses to myocardial infarction (MI). **A** Cardiac physiology of cohort 1 measured by echocardiography; values are average ± SEM. **B** Correlation heatmap of the top 5 protein candidates (ApoA1, haptoglobin, IgA, IL-17E, and TIMP-1) with the 6 cardiac physiology parameters (end systolic and diastolic dimensions and volumes, infarct wall thickness, and ejection fraction). **C** A representative correlation: ApoA1 negatively correlated with infarct wall thickness

Out of 165 unique proteins, 46 proteins correlated with at least one of the 6 cardiac physiology variables across D0 and MI D3 (all *p* < 0.05; primary echocardiography and proteome array data are provided in Supplemental Table 1). When we ranked significant proteins by *r* value, 24 proteins had *r* > 0.5 and 5 proteins had *r* > 0.6 for all 6 echocardiography variables. Figure 2 A shows the cardiac physiology at D0 and MI D3 in the retrospective cohort 1. The 5 proteins were apolipoprotein A1 (ApoA1), interleukin (IL)-17E/IL-25, immunoglobulin (Ig)A, haptoglobin, and tissue inhibitor of metalloproteinase-1 (TIMP-1). Figure 2 B shows the correlation heat map with r values for each of the 5 proteins with corresponding cardiac physiology variables. Figure 2C shows the representative correlation between ApoA1 and infarct wall thickness (IWT). These 5 proteins were further evaluated in a prospective study that evaluated by a secondary approach (immunoblotting) MI D3 and D7 plasma as well as MI D7 LVI in the same new set of mice. Supplementary Table 2 contains cardiac physiology and protein expression immunoblotting data for the prospective study cohort 2.

### ApoA1, IgA, IL-17E, and TIMP-1 Linearly Increased from D0 to D7 After MI

**Fig. 3:**
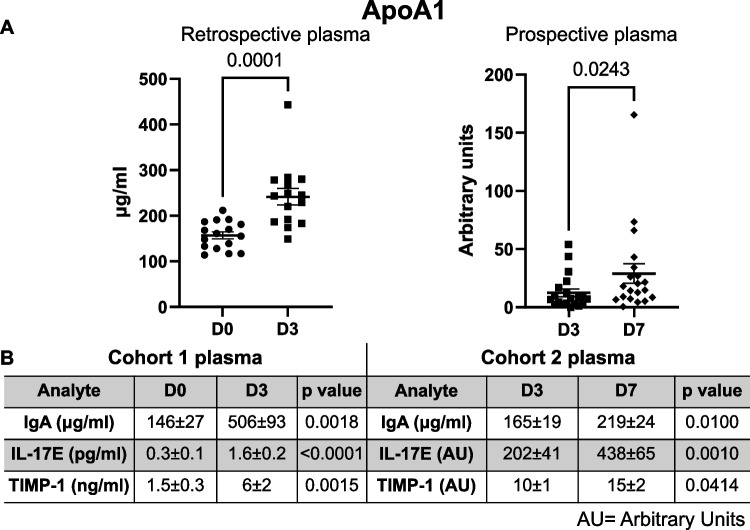
ApoA1, IL-17E, IgA, and TIMP1 increased after myocardial infarction (MI). **A** ApoA1 increased over the course of MI. ApoA1 increased from D0 to MI D3 in the retrospective plasma analysis (left). ApoA1 further increased from MI D3 to D7 in the prospective plasma analysis (right). **B** IgA, IL-17E, and TIMP-1 followed the same pattern as apoA1. The table summarizes protein expression in the plasma for both cohort 1 (D0 vs. D3) and cohort 2 (D3 vs. D7). Values are average ± SEM

In both the retrospective and prospective studies, plasma ApoA1, IgA, IL-17E, and TIMP-1 increased with MI (Fig. 3A). ApoA1 increased 1.5-fold from D0 to MI D3 in the retrospective study (Fig. 3A left) and increased 2.3-fold from D3 to D7 in the prospective study (Fig. 3A right). Similarly, IgA increased 3.5-fold from D0 to MI D3 and 1.3-fold from MI D3 to D7 (Fig. 3B and Supplementary Fig. 1); IL-17E increased 5.3-fold from D0 to MI D3 and 1.8-fold from MI D3 to D7 (Fig. 3B and Supplementary Fig. 1); and TIMP-1 increased 4.2-fold in plasma from D0 to MI D3 and increased 1.4-fold from MI D3 to D7 (Fig. 3B).

### ApoA1, IgA, IL-17E, and TIMP-1 Mirrored and Predicted Cardiac Physiology

**Fig. 4:**
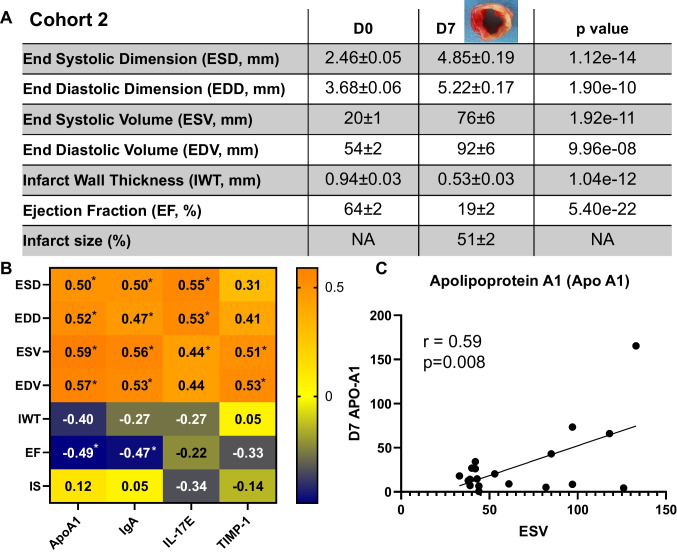
Validation in perspective cohort 2 for the 4 proteins, all of which mirrored cardiac physiology by regression analysis. **A** Cardiac physiology of cohort 2 measured by echocardiography. Values are average ± SEM. **B** Correlation heatmap of the top 4 plasma protein candidates (ApoA1, IgA, IL-17E, and TIMP-1) with the 6 cardiac physiology parameters (end systolic and diastolic dimensions and volumes, infarct wall thickness, and ejection fraction), as well as infarct size evaluated at necropsy. These 4 proteins offered extended predictability and mirrored cardiac physiology at MI D7. **C** A representative correlation: ApoA1 positively correlated with end systolic volume

Similar to the retrospective study for cohort 1, cohort 2 prospectively revealed significant MI specific cardiac physiology changes, indicated by increased dilation and infarct wall thinning and reduced ejection fraction (Fig. 4A). Of the 5 plasma proteins identified in cohort 1 as plasma predictors of adverse cardiac remodeling after MI at D3, 4 of them (ApoA1, IgA, IL-17E, and TIMP-1) showed extended predictability in the plasma from the MI D7 cohort (Fig. 4B). MI D3 plasma expression correlated with D7 plasma expression for ApoA1 (*r* = 0.65, *p* = 0.002), IgA (*r* = 0.74, *p* < 0.0001), and TIMP-1 (*r* = 0.45, *p* = 0.04). D7 LVI ApoA1 tissue expression correlated with cardiac physiology variables, including end systolic volume (Fig. 4C). Unlike plasma expression for the other 3 proteins (ApoA1, IL-17E, and TIMP-1), D7 LVI expression of IgA was the marker in the infarct that correlated best with cardiac physiology (Supplementary Fig. 2). Of note, ApoA1 was not detected in the LVI. IL-17E and TIMP-1 LVI tissue expression did not correlate with cardiac physiology. TIMP-1 LVI tissue expression was higher in females compared to males by 1.8-fold (Supplementary Fig. 2).

### Haptoglobin Peaked at MI D3 and Was a Time-Specific Marker of Cardiac Physiology

**Fig. 5:**
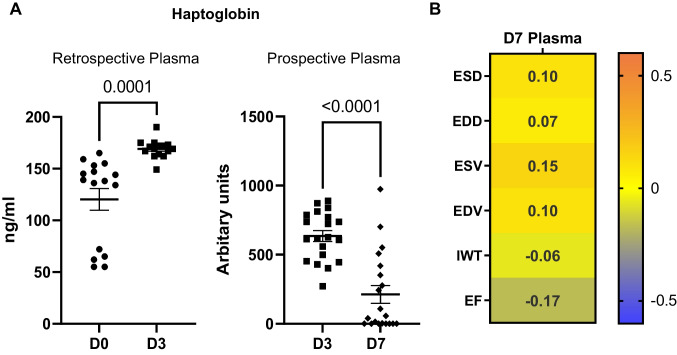
Haptoglobin increased early from D0 to MI D3 and decreased later from D3 to D7. **A** Haptoglobin expression peaked at MI D3. Haptoglobin increased from D0 to MI D3 in the retrospective plasma analysis (left). Haptoglobin decreased from MI D3 to D7 in the prospective plasma analysis (right). **B** Correlation heatmap of D7 plasma haptoglobin expression with D3 plasma expression and the D7 cardiac physiology variables

Haptoglobin peaked in the plasma at MI D3, giving an inverted U-shaped expression curve. Haptoglobin increased 1.4-fold from D0 to MI D3 in the retrospective analysis (Fig. 5A left) and decreased 3.0-fold from MI D3 to D7 in the perspective analysis (Fig. 5A right). Plasma haptoglobin expression correlated with cardiac physiology at MI D3 for the retrospective study, but not the prospective study, indicating haptoglobin was a time specific marker of MI (Fig. 5B). Haptoglobin expression was not detected in the LVI.

## Cytokine-Cytokine Receptor Interaction Was the Most Enriched KEGG Pathway

**Fig. 6:**
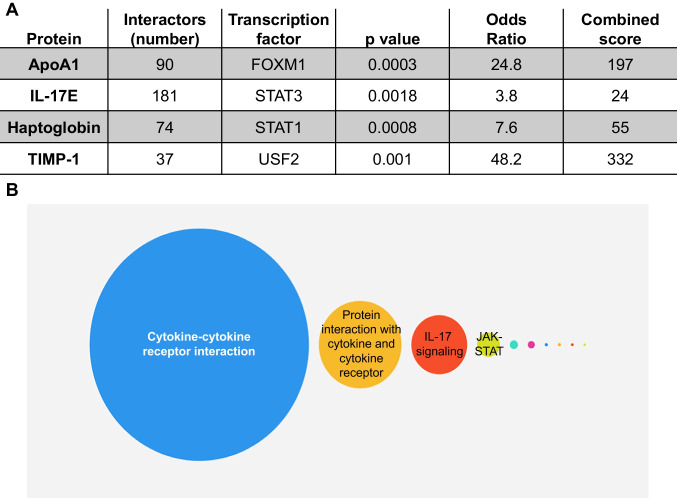
Human glycoproteomic analysis showed that ApoA1, IL-17E, TIMP-1, and haptoglobin all signaled primarily through cytokine-cytokine receptor signaling after MI. **A** Protein–protein interactions among each identified plasma marker and the 999 other glycoproteins measured in the array showed enrichment of FOXM1 (ApoA1), STAT1 (IL-17E), STAT3 (haptoglobin), and USF2 (TIMP-1) transcription factors. **B** Cytokine-cytokine receptor signaling was the most enriched pathway as shown in the bubble chart

By human glycoproteomic analysis of protein–protein interactions, ApoA1 correlated with 90 other glycoproteins, IL-17E correlated with 181 other glycoproteins, haptoglobin correlated with 74 other glycoproteins, and TIMP-1 correlated with 37 other glycoproteins. By enrichment analysis of the proteins that interacted with the candidates, the most enriched transcription factor for apoA1 was forkhead box M1 (FOXM1), for IL-17E was signal transducer and activator of transcription (STAT) 3, for haptoglobin was STAT1, and for TIMP-1 was upstream transcription factor 2 (USF2; Fig. 6A). By KEGG human pathway analysis, ApoA1, IL-17E, haptoglobin, and TIMP-1 were all enriched for the cytokine-cytokine receptor interaction pathway (Fig. 6B). The top 25 proteins that correlated with apoA1, IL-17E, haptoglobin, and TIMP-1 are shown in Supplementary Fig. 3. Supplementary Fig. 4 shows the individual KEGG pathways enriched for each of the 4 plasma proteins.

## Discussion

**Fig. 7:**
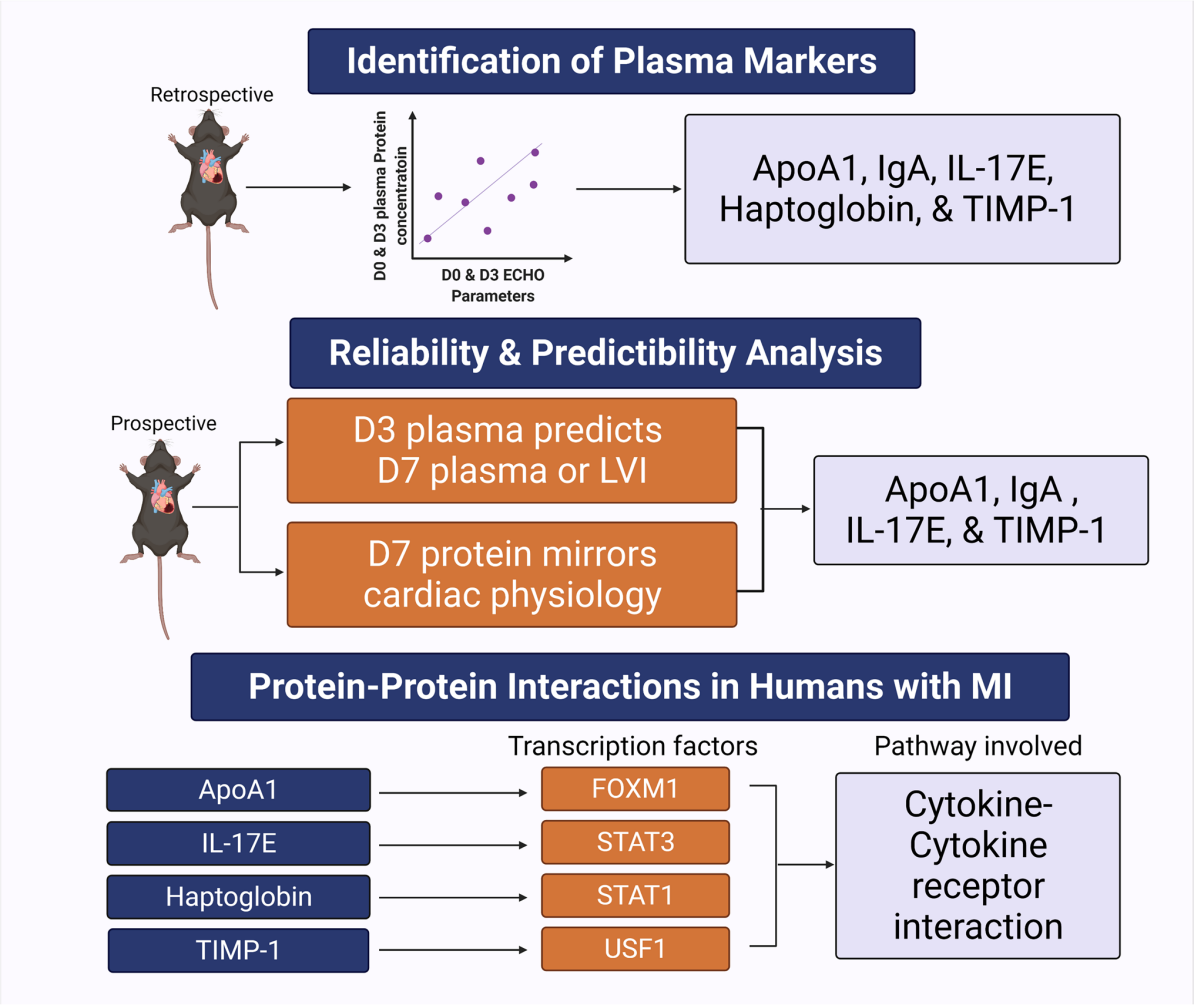
We identified four plasma proteins — ApoA1, IgA, IL-17E, and TIMP-1 — involved in MI wound repair that mirror current and predict the future continuation of adverse cardiac remodeling. Created with BioRender.com

The objectives of this study were to (1) identify the plasma proteins at MI D3 that best mirror LV wall thinning and dilation in a mouse MI model, (2) validate candidates in a second cohort of mice for the ability to predict cardiac remodeling at MI D7, and (3) evaluate translation to humans with MI. Five proteins were identified in the MI D3 plasma that reflected echocardiography: ApoA1, Haptoglobin, IgA, IL-17E, and TIMP-1. Of these proteins, ApoA1, IgA, and IL-17E mirrored current and predicted future adverse cardiac remodeling. Haptoglobin was a time specific indicator. The plasma markers identified were tested across three different time points (D0 and MI D3 and D7), in two different sources (plasma and infarct), and at two different age groups (4 and 20 months) in two different cohorts of mice. Thus, the results reflect cardiac remodeling that spans cardiac wound healing phases and age, which may help to translate these findings as clinical markers of adverse remodeling. In the human cohort, cytokine-cytokine receptor interaction was the most enriched pathway, with FOXM1, STAT1, STAT3, and USF2 being the most enriched transcription factors. Combined, our results revealed that plasma could be used to reflect present and predict future extent of LV infarct wall thinning and dilation (Fig. 7).

ApoA1 is a major component of high-density lipoprotein (HDL) cholesterol, which transports excess cholesterol from peripheral tissues to the liver. HDL decreases early in MI and gradually increases over time as a secondary protective mechanism [46]. The increase in ApoA1 may indicate increased liver function after MI [47]. ApoA1 was previously identified as a better predictor for ischemic heart disease and cardiovascular mortality than HDL, low-density lipoprotein, or apolipoprotein B [48]. ApoA1 positively correlates with HDL and negatively correlates with c-reactive protein. The Apolipoprotein-related MOrtality RISk study (AMORIS) showed that increased risk of fatal MI strongly correlated with ApoB/ApoA1 ratio, indicating ApoA1 serves protective roles prior to MI [49]. ApoA1 before MI is associated with risk of MI, and the role of ApoA1 after MI could follow a similar course as HDL.

Similar to our observations in mice, increased IL-17E/IL-25 also associates with severity of coronary artery disease (CAD) in humans[50]. Macrophages and T lymphocytes are major sources of IL-17E, and both macrophages and T lymphocytes increase under a variety of ischemic conditions, including acute MI and unstable and stable angina pectoris [50]. IL-17E promotes TH2 cytokine responses, and inhibition of IL-17E in cancer increases macrophage and T lymphocyte numbers by inhibiting apoptosis and promoting cellular proliferation [51, 52]. IL-17E expression in patients with CAD correlates with TNFα, IL-6 levels, and Gensini score in atherosclerosis, with a zero score indicating no atherosclerosis [50]. IL-17E promotes angiogenesis by increasing vascular endothelial growth factor signaling in endothelial cells [53]. IL-17E could potentially be associated with increased inflammatory response and fibrosis. Our understanding of the role of IL-17E in MI is incomplete and needs further study.

In line with our findings, others have shown that IgA increases with MI [54, 55]. We extend these past observations to show linearity in the increase of plasma IgA level over the course of MI remodeling in mice. Elevated plasma IgA is a marker of previous MI, indicating temporal sustainability in humans [55]. Increased IgA in MI could be due to increased B-cell mediated inflammation. Alarmins activate B-cells in MI to induce atherosclerosis by activating plasma cells to produce immunoglobulins [56]. Targeted B-cell therapy has been suggested in atherosclerosis and various cardiovascular diseases as a potential treatment option [57, 58]. B-cell depletion using monoclonal CD-20 antibody resulted in better MI remodeling through reduced monocyte recruitment [59, 60].

TIMP-1 is an endogenous inhibitor of matrix metalloproteinases (MMPs), including MMP-9 [61]. In a human study with 389 males undergoing coronary angiography, TIMP-1 was identified as the only biomarker that could independently predict all-cause mortality and MI [62]. In that study, lower plasma TIMP-1 after MI yielded improved survival rates. TIMP-1 and MMP-9 are documented indicators of cardiac remodeling after MI [63]. TIMP-1 and IL-8 have been identified as markers that indicate ventricular fibrillation in MI patients [64]. The biological function of TIMP-1 may have a U-shaped curve, as animal studies with overexpression of TIMP-1 also show protection [65]. Higher TIMP-1 LVI expression in females than males is in line with previous studies showing estrogen controls TIMP-1 expression [66]. TIMP-1 expression was decreased in hearts of ovariectomized volume overloaded female rats and was restored with the administration of estrogen [66]. Therefore, TIMP-1 demonstrates sex-dependent regulation.

Haptoglobin is a plasma protein that binds to hemoglobin, and increased binding occurs in response to immune activation. Haptoglobin increases after MI and elevated haptoglobin is a known risk factor for MI and congestive heart failure [67–69]. The AMORIS study revealed that increased haptoglobin correlated to a higher risk ratio for MI, irrespective of total cholesterol levels [67]. We observed a time specific change in haptoglobin expression, with an inverted U-shaped pattern in mice over the first week of MI remodeling, which could be due to inflammation going down after MI D3 as haptoglobin is a known positive acute phase protein [70]. As such, haptoglobin is likely a better early diagnostic and prognostic marker of MI.

In the validation perspective cohort 2, four identified markers (ApoA1, IgA, IL-17E, and TIMP-1) extended from MI D3 to D7 in terms of ability of plasma concentrations to mirror current cardiac physiology status. This was important to assess because information on valid clinical equivalence for mouse MI timepoints is not available, and markers that can predict cardiac physiology across MI remodeling timepoints will be valuable for translation. In contrast, haptoglobin has a small window of predictability, making it easy to miss the optimum evaluation time. Identification of the MI timepoint equivalence in mouse vs. human is lacking, as is the range of inter-person variability in timing. Therefore, it would be difficult to know a potential optimal time of evaluation for humans.

Studies are also warranted to determine if these markers improve early identification of patients vulnerable for later death or development of congestive heart failure or improve our ability to assess efficacy of therapy. Given that hypertension and family history of heart disease, as well as diabetes and obesity, are major comorbidities in humans with MI, future translation of these results to clinical application will need to take these variables into consideration.

Bioinformatics of the human cohort revealed cytokine-cytokine receptor pathway as the most enriched signaling pathway for all 4 proteins (IgA was not measured in the human cohort). While seeing representation of the inflammatory response was expected for MI samples, having every single identified marker being associated with the inflammatory response was not expected. FOXM1, STAT3, STAT1, and USF2 were the transcription factors associated with ApoA1, IL-17E, haptoglobin, and TIMP-1 respectively. FOXM1 is required for cardiomyocyte development and has cardioprotective properties [71, 72]. Similar to FOXM1, activation of STAT3 signaling in MI has protective actions to inhibit inflammation [73–75]. Rapamycin and empagliflozin both attenuate MI remodeling through actions on cell death and both activate STAT3 signaling [76, 77]. STAT1 inhibits autophagy and is detrimental in MI remodeling by promoting inflammation [78, 79]. While information on the role of USF2 in MI is limited, USF2 is one of the top 5 upregulated transcription factors with larger transcription regulator network associated with differentially expressed genes in MI, along with STAT3 [80]. USF2 is associated with iron overload and regulates hepcidin expression, which increases in the LV remote region after MI [81]. These transcription factors, therefore, are directly and indirectly connected with MI remodeling.

## Conclusion

Our results revealed that MI shifts the plasma proteome to mirror and reflect adverse cardiac remodeling, with ApoA1, IL-17E, IgA, haptoglobin, and TIMP-1 serving as possible indicators of ongoing active cardiac remodeling. Out of the five, ApoA1, IL-17E, IgA, and TIMP-1 have an extended window for predicting cardiac physiology after MI, while the window is narrow for haptoglobin. All markers signal primarily through the cytokine-cytokine receptor interaction pathway, indicating inflammatory response is a common denominator. Clinical evaluation of these markers may help to improve early identification of patients vulnerable for later death or development of congestive heart failure or to assess efficacy of therapy.

## Supplementary Information

Below is the link to the electronic supplementary material.

Supplementary file1 (PPTX 120084 KB)

Supplementary file2 (XLS 88 KB)

Supplementary file3 (XLS 37 KB)

## Abbreviations

ApoA1: Apolipoprotein A1
D: Day
EDD: End diastolic dimension
EDV: End diastolic volume
ESD: End systolic dimension
ESV: End systolic volume
F: Female
FOXM1: Forkhead Box M1
HDL: High-density lipoprotein
IgA: Immunoglobulin A
IL: Interleukin
IRB: Institutional review board
IS: Infarct size
IWT: Infarct wall thickness
LVI: Left ventricular infarct
M: Male
mHART: Mouse Heart Attack Research Tool
MI: Myocardial infarction
MMP: Matrix metalloproteinase
STAT: Signal transducer and activator of transcription
TIMP-1: Tissue inhibitor of matrix metalloproteinases-1
TTC: 1% 2,3,5-Triphenyl tetrazolium chloride
USF2: Upstream transcription factor 2
